# Deciphering Proteomic Signatures of Early Diapause in *Nasonia*

**DOI:** 10.1371/journal.pone.0006394

**Published:** 2009-07-28

**Authors:** Florian Wolschin, Jürgen Gadau

**Affiliations:** 1 School of Life Sciences, Arizona State University, Tempe, Arizona, United States of America; 2 Department of Biotechnology, Chemistry, and Food Science, Norwegian University of Life Sciences, Ås, Norway; University of Oldenburg, Germany

## Abstract

Insect diapause is an alternative life-history strategy used to increase longevity and survival in harsh environmental conditions. Even though some aspects of diapause are well investigated, broader scale studies that elucidate the global metabolic adjustments required for this remarkable trait, are rare. In order to better understand the metabolic changes during early insect diapause, we used a shotgun proteomics approach on early diapausing and non-diapausing larvae of the recently sequenced hymenopteran model organism *Nasonia vitripennis*. Our results deliver insights into the molecular underpinnings of diapause in *Nasonia* and corroborate previously reported diapause-associated features for invertebrates, such as a diapause-dependent abundance change for heat shock and storage proteins. Furthermore, we observed a diapause-dependent switch in enzymes involved in glycerol synthesis and a vastly changed capacity for protein synthesis and degradation. The abundance of structural proteins and proteins involved in protein synthesis decreased with increasing diapause duration, while the abundance of proteins likely involved in diapause maintenance (e.g. ferritins) increased. Only few potentially diapause-specific proteins were identified suggesting that diapause in *Nasonia* relies to a large extent on a modulation of pre-existing pathways. Studying a diapause syndrome on a proteomic level rather than isolated pathways or physiological networks, has proven to be an efficient and successful avenue to understand molecular mechanisms involved in diapause.

## Introduction

Diapause is an alternative life-history trait that enables an individual to endure unfavorable environmental conditions e.g. drought and cold. We here refer to diapause as a syndrome as it is characterized by a set of metabolic features that are closely associated and, in combination, can result in a drastically altered physiology [1], [2]. Diapause can be facultative (elicited by direct environmental cues) or obligatory (independent of temporary environmental changes, probably evolved from facultative diapause) and it entails a state of decreased inactivity thought of as developmental arrest [3]. In insects, diapause requires an array of sophisticated metabolic adjustments and can appear during development (developmental diapause) and adulthood (reproductive diapause). Thus, instead of a static state it can be viewed as a dynamic process that is divided in early, intermediate, and late phases.

The remarkable characteristics of diapause have led to an ever-increasing amount of research focusing on this topic. Studies in *C. elegans* and *Drosophila* were able to unravel some of the molecular details of reproductive diapause due to the availability of a whole genome sequence [4]–[6]. Hormones influence diapause and it appears that insulin signaling plays a major role in diapause regulation, which could potentially explain a known connection between diapause and longevity [6]–[9]. Recently, diapause has also been implicated in the evolution of castes in social wasps pointing out that the molecular building blocks of diapause might have been used to regulate reproductive division of labor in some insect societies [10].

However, our understanding of the molecular mechanisms involved in generating, maintaining, and breaking diapause is still highly fragmentary (Denlinger 2002). This is especially true for developmental diapause since genome sequences for model organisms undergoing this form of diapause were missing until recently.

Also, it is unclear to what extent metabolic patterns are conserved across species that exhibit the same or different diapause syndromes (facultative or obligatory, developmental and reproductive). Thus, there is an ongoing need for comparative studies that reveal the molecular details underlying diapause.

To this end, unbiased large-scale studies are particularly useful since they are not restricted by prior assumptions and can, in a single study, reveal several diapause characteristics [11]–[14]. However, to make full use of available molecular tools, a sequenced genome is a pre-requisite. *Nasonia vitripennis*, a parasitic solitary wasp that can go into developmental diapause, is emerging as a new insect model system especially due to the availability of a sequenced genome [15] and functional genomic tools like RNAi [16]. This makes it a suitable candidate for large-scale molecular studies aiming at deciphering diapause characteristics.

Diapause in *Nasonia* has been shown to be maternally induced and it probably serves as an adaptation to the temperate life zone (Northern America, Europe, and Northern Asia) since *Nasonia* hosts (flesh fly pupae) are unavailable during winter. Multiple external (e.g. temperature, photoperiod, host) and internal (e.g. age of mother, number of eggs laid) factors that act on any egg-laying female are known to induce diapause in her offspring [17]–[20]. A factor of unknown structure is thought to be passed on from the mother to the offspring leading to an adaptation of metabolism that finally results in the observed diapause syndrome in the last larval instar [19], [20]. In this work, we focused on the proteomic changes during early diapause in *N. vitripennis* for three major reasons: 1. early diapause sets the stage for all processes that follow and can be thought of as a metabolic switch point. This is especially true for *Nasonia vitripennis*, a species that at day 6 of its life reaches a crucial stage in larval development. At this stage, it will either initiate diapause or pupate; 2. proteomic approaches do not reach the depth of transcriptome studies. However, they are especially well suited to study the intricacies of diapause because they reflect the physiological state in a more direct way than RNA studies; 3. the availability of an annotated genome sequence for *N. vitripennis* enabled us to maximize output.

Our results reveal novel insights into the metabolic processes during the early stages of diapause in *Nasonia* and corroborate the ubiquity of some diapause-associated patterns in insects and copepods. They can now serve as a foundation for future studies that will focus on specific metabolic processes during the induction, maintenance, and breakage of diapause in *Nasonia*.

## Methods

### Collection of *Nasonia*

In all experiments *Nasonia vitripennis* (ASYMCX) females (all sisters from a highly inbred line) were set as virgins on *Sarcophaga bullata* hosts. This procedure guaranteed that only closely related larvae of one sex (males) were produced (as a Hymenoptera, *Nasonia* exhibits a haplo- diploid sex determination mechanism).

Two quality control steps ensured the accurate collection of diapausing larvae:

1. Diapausing larvae were only collected after 100% of the previous batch of their mother's offspring (a given virgin female) had entered diapause. In short, females were provided individually with host pupae for 24 hours every other day for 14–20 days. The parasitized hosts were then transferred into empty vials in which the wasp larvae completed development. After 10 days the hosts were opened and the percentage of diapausing larvae was determined (non-diapausing larvae would be well into a pupal stage at that time). As soon as all larval offspring of a female entered diapause, females were provided with new hosts in order to produce larvae suitable for analysis. Diapausing larvae were collected 6, 7, and 8 days after the female was provided with the host pupae (DP6-8).
2. Control samples (larvae in unopened pupae) from the final re-hosting (see above) were monitored after one week to ensure that 100% of the larvae went into diapause.

Three *Nasonia* larvae (all from one host) were pooled per sample in order to decrease host-dependent variation (under the conditions used a given female lays only one batch of eggs into one host pupa).

Non-diapausing larvae were collected 6 days after a female was given an individual host for 24 hours. Three larvae produced by one female were pooled for analysis.

Treatment to induce diapause included exposure to constant darkness and 4°C. Control females were kept under constant light at room temperature.

### Protein extraction, quantification, and digestion

Samples consisting of three larvae were homogenized in 150 µl of protein extraction buffer (50 mM tris pH 8.5, 2% SDS, 5% beta-mercaptoethanol, 0.15 M NaCl, 30% glycerol). Samples were then vortexed vigorously, boiled at 95°C for 5 min, vortexed again, and centrifuged for 2 min at 10,000 rcf. The supernatant was subjected to methanol/chloroform precipitation as described in [21].

Proteins were redissolved in 50 µl of buffer containing 50 mM tris pH 8.5, 6 M urea, 2 M thiourea, 0.15 M NaCl, 1 mM CaCl_2_. Consecutively, 150 µl of buffer (50 mM tris pH 8.5, 0.15 M NaCl, 1 mM CaCl_2)_ were added, samples were spun at 10,000 rcf for 2 min, and the supernatant was used for further analysis. The Bradford assay was used to determine protein concentration [22] and 40 µg per sample were subjected to digestion over night at 30°C with 1 µg of trypsin in digestion buffer (50 mM tris pH 8.5, 0.15 M NaCl, 1 mM CaCl_2_).

Peptide desalting was performed the next day as described before [23], [24].

### LC-MS/MS Analysis

Dried peptides (from 10 µg protein) were dissolved in 5% acetonitrile, 2% TFA and used in a non-targeted LC-MS/MS analysis. Peptides were separated on a picofrit column (75 µm ID, New objective, Woburn, USA) using a 105 min gradient ranging from 95% A (0.1% formic acid, 99.9% H_2_O) to 80% B (0.1% formic acid, 99.9% acetonitrile) followed by a 15 min equilibration step. Peptides were eluted from the reversed phase µLC column directly into an LTQ mass spectrometer (Thermo, San Diego, USA) and the following settings were used: isolation window:3 m/z, collision energy: 35, and activation time: 30 ms. MS^2^ spectra were recorded for the five most abundant peaks in each MS survey spectrum. Using the open source search tool OMSSA (version 2.0.0) [25] the spectra were matched against an *N. vitripennis* sequence database retrieved from NCBI (http://www.ncbi.nlm.nih.gov/) containing additional trypsin and keratin sequences. The following filtering criteria were used: 0.8 Da fragment tolerance, 0.8 Da precursor tolerance, maximum of 2 missed cleavages, only tryptic sequences allowed, initially ten possible peptide hits per spectrum reported then filtered to one peptide hit per spectrum, variable modifications: methionine oxidation, deamidation of N and Q. Acceptance threshold: e≤0.1.

The overlap of proteins was calculated by comparing accession numbers resulting from analyses of the different sample groups (NDP6, DP6-8). The output was displayed in a diagram capable of comparing the overlap of four groups (a 4-way Venn diagram adapted from [26]). One database entry hit is characterized by at least one peptide at e≤0.1.

### Statistical evaluation

Statistical evaluation of the data was essentially carried out as described in [24] with the following modifications:

Individual spectral count (count per peptide) was divided by the overall spectral count corrected for the number of spectra belonging to host proteins of the same sample. This was done because it was observed that non-diapausing samples had a tendency to produce more hits to host proteins. Thus, the spectral count for all identified host proteins was subtracted from the overall spectral count of the sample to minimize competition effects. Criteria for quantification: 1. hits were only accepted at a threshold of two peptides e≤0.1; 2. spectral count of at least 3 in at least 4 of the samples of one sample set was required for acceptance. A decoy database with reversed sequences was used to ensure that the false discovery rate for peptides was below 0.95%.

For hierarchical clustering analysis the corrected spectral count values for each protein were z-transformed ((individual value - standard deviation)/average value). TM4 version 4.1.01 was used for computing the HCA (Pearson correlation coefficient, complete linkage clustering). For simplification purposes we excluded protein isoforms with highly similar abundance patterns to the ones displayed here.

## Results

Following day six, *Nasonia* development diverges into either pupation (favorable conditions) or diapause (unfavorable conditions). We here focused at proteomic changes during this crucial switchpoint. Sampling resulted in four distinct groups: 6-day-old non-diapausing larvae (NDP6), and 6-8 day-old diapausing larvae (DP6, DP7, and DP8). See methods section for details.

### 1. Shared proteins/peptides

**Figure 1:**
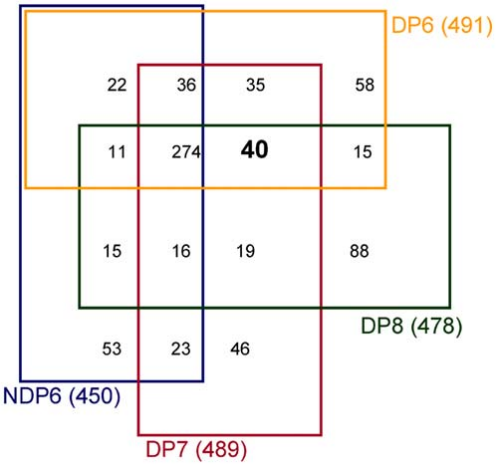
Overlap between proteins/peptides found in non-diapausing and diapausing larvae. Shown is the overlap of protein/peptide hits for all collected specimens (NDP6, DP6-8) in a 4-way Venn diagram. DP6-8: diapausing larvae collected at days 6–8 (orange, red, and green, respectively). NDP6: non-diapausing larvae collected at day 6 (blue). Hits only identified in the diapause-stages are highlighted in bold.

Most of the identified peptides were found in all sample groups and there was little evidence for diapause-exclusive expression (40 database entry hits. One database entry hit is characterized by the identification of at least one peptide at e≤0.1 matching to an accession number in the database. See S1 for the number of peptides corresponding to the respective database entry hits). This list decreases to 37 entries when redundancy and a partial sequence entry are dismissed. The overlap is exemplified in a 4-way Venn diagram [Figure 1, see also supplementary table S1 for all database entries and supplementary table S2 for individual peptides]. Several peptide hits match to proteins that are known to be of high importance during diapause of other invertebrate species e.g. proteins involved in fat (long-chain-fatty-acid-CoA ligase, 3-2-trans-enoyl-CoA isomerase, enoyl-CoA hydratase/isomerase family, 3-hydroxyacyl-CoA dehydrogenase) and sugar (trehalose 6-phosphate synthase, triosephosphate isomerase) metabolism [supplementary table S1]. Note that the database entries that were exclusively identified for diapausing larvae or for individual diapausing stages could be present below the detection limit in non-diapausing larvae.

### 2. Heatmap of quantifiable proteins

**Figure 2:**
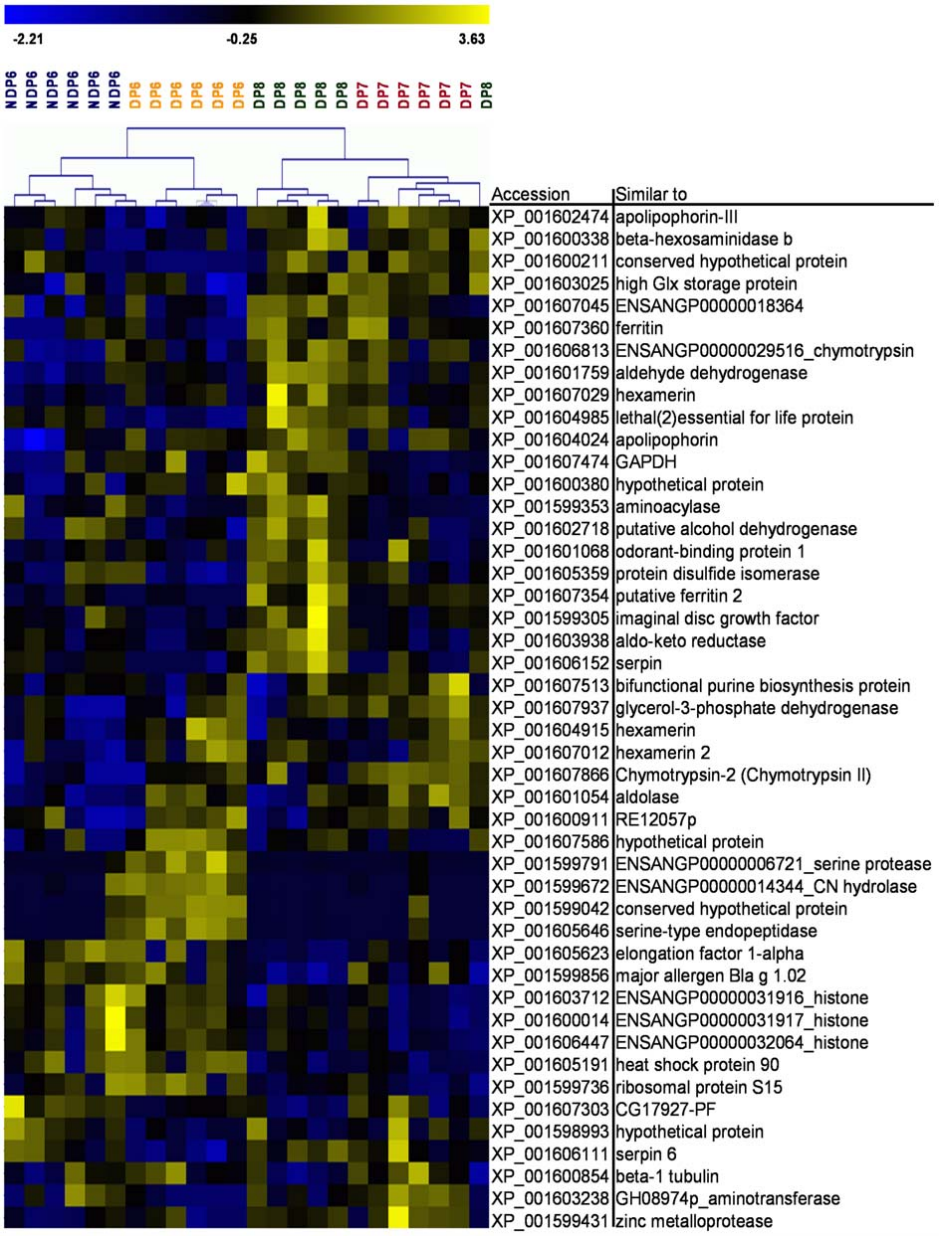
Heatmap and hierarchical clustering of selected proteins. This protein expression map (heatmap) visualizes protein abundance differences between individual samples. Proteins shown were selected out of all quantifiable proteins by a Kruskal Wallis test. This procedure resulted in proteins that showed significant (bootstrap corrected p-value cutoff: p≤0.1) differences over the four sample groups (NDP6 and DP6-8). The abundance increases from blue to yellow. Samples were grouped based on Pearson correlation and full linkage clustering. Rows indicate individual proteins, columns indicate individual samples. Comparisons of protein abundances are valid within rows but not within colums. DP6-8: diapausing larvae collected at days 6–8 (orange, red, and green, respectively). NDP6: non-diapausing larvae collected at day 6 (blue).

Proteins were quantified using spectral count as described in the methods section. Quantified proteins were subjected to a Kruskall-Wallis test to determine proteins that differed in their abundance between any of the sampling groups and a bootstrap algorithm was employed in order to adjust the p-value cutoff [24]. Hierarchical clustering analysis on proteins with p≤0.1 was used to visualize homogeneity of the samples and variances between groups [Figure 2]. The data show generally good separation of the four groups (NDP6 and DP6-8) due to greater homogeneity within groups compared to between groups. Differences between diapausing and non-diapausing stages as well as within diapausing stages are apparent by the clustering trees. Protein descriptions [Figure 2] following an underscore were obtained through BLAST search (e-value≤1 e^−40^). Outliers (samples not clustered within their respective group) possibly represent heterogeneity within a population (one DP6 in a cluster of NDP6 samples) and overlap between sample groups (one DP8 in a cluster of DP7 samples). Due to a 24 h incubation period (the time females were exposed to host pupae), overlap between the groups DP6 and DP7 or between DP7 and DP8 can not be entirely excluded. This can explain the DP8 outlier.

Our results indicate a shift from proteins involved in structure and protein turnover (histone, ribosomal protein, elongation factor, proteases) to enzymes and proteins necessary for diapause maintenance (metabolic enzymes, ferritin) from DP6 to DP8.

### 3. Selected protein groups affected by diapause

#### 3.1 Protein synthesis

The abundance of elongation factor 1, ribosomal protein S15, and a histone-like protein was found to be high at day 6 compared to day 8 of diapausing larvae suggesting a progressive decline of the levels of these proteins during initial diapause stages [Mann Whitney U-tests for DP6 and DP8, p≤0.05, see also Figure 2]. However, no abundance changes were observed for another ribosomal protein (S7) and translation elongation factor 2.

#### 3.2 Protein degradation

A set of five proteases/peptidases and two potential serin protease inhibitors (serpins) revealed a complex pattern of proteolytic capacities.

Notably, one of the proteases showed high levels only in diapausing larvae day 6 (the last day before pupation or ongoing diapause, XP_001605646) [Figure 2].

#### 3.3 Cryoprotection

Three enzymes that can be related to the synthesis of glycerol, (aldolase, glyceraldehyde-3-phosphate-dehydrogenase, and glycerol-3-phosphate dehydrogenase), were found to have varying abundance patters for the different groups [see Figure 2 and supplementary table S1]. In addition, when selection criteria for quantification were made less stringent, two additional enzymes that are putatively involved in glycerol metabolism (phosphoglucomutase and triose-phosphate isomerase) were found at higher levels during at least one of the diapausing stages (data not shown).

#### 3.4 Proteins involved in damage prevention

**Figure 3:**
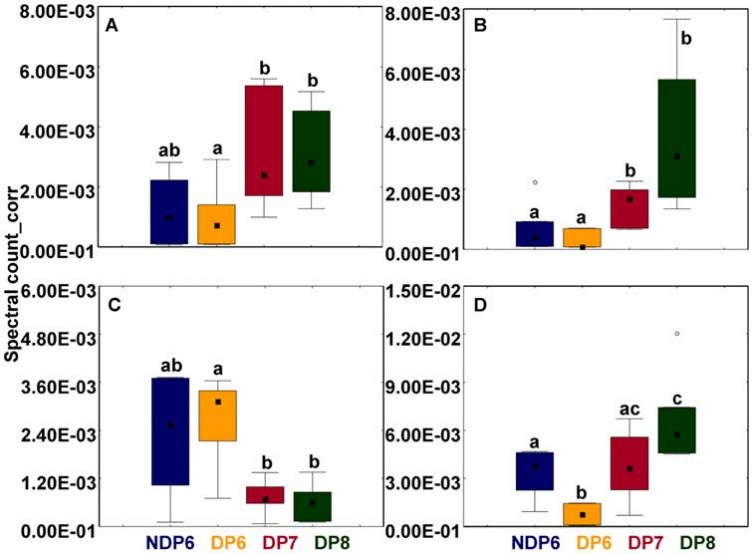
Abundance changes in putative stress-response proteins during early diapause in *Nasonia*. Abundance levels of proteins similar to Ferritin, Ferritin 2 (panels A and B, respectively), HSP90, and LEFL, a member of the HSP20 family (panels C and D, respectively) represented in box plots (median and 25–75% confidence interval). Letters denote significant differences (Mann Whitney U-test, p≤0.05, n = 6 per group). Open circles represent outliers. Spectral count_corr: individual spectral count normalized to total spectral count. DP6-8: diapausing larvae collected at days 6–8 (orange, red, and green, respectively). NDP6: non-diapausing larvae collected at day 6 (blue).

We found that both ferritin subunits are upregulated in diapausing *Nasonia* larvae [Figure 3A and B], while the pattern of two proteins that belong to the family of heat shock proteins was more variable. At day 6, heat shock protein 90 abundance levels were comparable between diapausing and non-diapausing larvae, but its levels dropped with increased diapause duration. In contrast, the abundance of a heat shock protein 20 family member (lethal essential for life) was low at the beginning of diapause and increased over time [Figure 3C and D].

#### 3.5 Storage proteins

Five storage proteins of this category were identified and quantified [Table 1, figure 2]. We found upregulation, downregulation, and steady levels for this protein family.

**Table 1:** Storage protein abundance differences between groups.

Protein predicted/similar to	Accession	NDP6	DP6	DP7	DP8
High Glx storage protein	XP_001599110	a	a	a	a
High Glx storage protein	XP_001603025	a	ab	b	ab
Hexamerin 2	XP_001607012	a	b	b	b
Hexamerin	XP_001604915	a	b	ab	ab
Hexamerin	XP_001607029	a	ab	ab	b
Apolipophorin	XP_001604024	a	b	ab	b
Apolipophorin-III	XP_001602474	a	a	b	ab

Different letters indicate statistically different expression (Mann Whitney U-test, p≤0.05, n = 6 per group).

## Discussion

The alternative life-histories of *Nasonia*, diapause vs non-diapause, are associated with clear protein abundance differences. However, most of the identified peptides overlap between stages, suggesting that *Nasonia* exerts diapause by mainly varying the abundance of proteins that are already present in non-diapausing larvae.

Our results imply that a metabolic shift from reconstruction to maintenance occurs during these early steps of diapause. High levels of proteins involved in replication/transciption (histones) and translation (ribosomal protein, translation factor) at day 6 are followed by an increase of metabolic enzymes (e.g. GAPDH, GDH, aldolase) and maintenance proteins (ferritin, some hexamerins) during stages DP 7 and DP 8.

Lower levels of ribosomal proteins and elongation factors during diapause agree well with previous reports on diapause protein production capacity and cell growth [27]. Similarly, there is an established link between histone expression and replication/translation [28]. Thus, the decrease of histone levels with time in diapause observed in this study points to an impairment/downregulation of these functions. Adding to the idea of organismal restructuring during early diapause, we found an actin to be highly abundant early in diapause. This observation fits with earlier reports on the modification of actin expression in diapausing stages of *Culex pipiens* [29], in which it was speculated that actin plays a role in modification of the cytoskeleton during early diapause.

Another group of proteins that showed varying abundance levels during the transition phase are proteases. We found that several proteolytic enzymes and protease inhibitors have different abundance patterns in non-diapausing and diapausing larvae. This result corroborates previous reports showing that some proteases and digestive enzymes are closely linked to diapause [30], [31]. It appears that the digestive system and the overall proteolytic capacity have to undergo major changes in order to cope with the change in nutrient availability and to help with restructuring the entire metabolism.

Most notably, a peptidase showed very high relative levels of abundance at DP6 (one of the NDP6 samples falls into the same category, probably representing an intermediate or diapause larvae in the control group). This feature was shared with two hypothetical proteins and a CN hydrolase-like protein and suggests a co-regulation that is of importance during early diapause.

No comparative data is available for the hypothetical proteins. However, the CN hydrolase-like protein might be involved in the recycling of building blocks. CN hydrolases catalyze the hydrolytic cleavage of carbon-nitrogen bonds and their action often yields free ammonia that, in turn, might be used to synthesize a pool of new amino acids [32], [33].

Following a decrease in the overall capacity of cell growth (DP6), metabolic mechanisms that are potentially essential for diapause maintenance are put into place starting with DP7.

Glycerol synthesis is commonly associated with diapause [34]–[36]. Under conditions of normal development glycerol is mainly used to make triacylglycerols and phospholipids. However, elevated production of glycerol is thought to increase the cold-hardiness in a variety of organisms [34]–[40] and glycerol levels were shown to be host- and temperature-dependent in *Nasonia* [41].

In our study we monitored protein abundance levels, which do not necessarily directly correspond to changes in enzymatic activity. However, it is intriguing that we observed diapause-dependent abundance patterns for proteins that are potentially involved in glycerol production. GDH abundance levels for DP6, DP7, and DP8, were not statistically different from one another, while levels for NDP6 did differ significantly from levels at DP7 but not from levels at DP6 or DP8. We propose two possible reasons as to why GDH abundance appears to reach a maximum before DP8: 1. enzyme levels might oscillate during diapause or 2. glycerol production might only be increased during a very limited timeframe. In this context it is interesting to review the relationship between GDH and GAPDH. If glycerol synthesis is increased, there will be less fuel available for GAPDH and subsequent steps in glycolysis. Thus, it might be important for the organism to either switch back and forth between emphasizing one or the other branch and/or to limit glycerol synthesis to a narrow time window in order to facilitate sufficient glycolysis.

**Figure 4:**
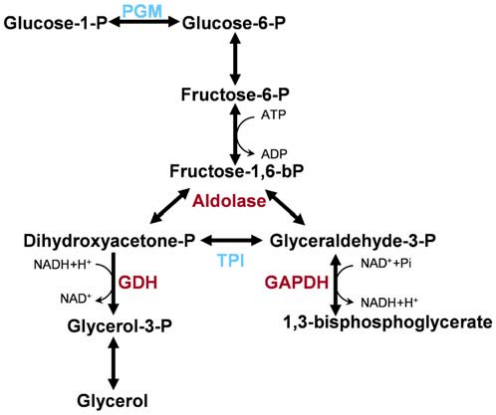
Suggested pathway for glycerol synthesis in *Nasonia.* Proteins putatively involved in glycerol production and glycolysis had significantly different abundance levels between groups (Kruskal Wallis and Mann Whitney U-tests, p≤0.05) leading us to suggest this pathway for glycerol synthesis in *Nasonia* (enzymes in red). Enzymes in blue were detected at higher levels in diapausing stages but did not pass all criteria for quantification. GAPDH: glyceraldehyde-3-phosphate-dehydrogenase, GADH: glycerol-3-phosphate dehydrogenase, PGM: phosphoglucomutase.

Drawing from our results and from older data on diapause in *Bombyx mori* [35] we propose a possible pathway for glycerol production in *Nasonia* [Figure 4].

Other proteins that might directly or indirectly protect the organism from damage are ferritin and heat shock proteins. Ferritin is known to be involved in protection of molecules from oxidative damage by scavenging oxygen radicals. However, it has also been implicated in the control of cell proliferation by regulating the availability of iron [42]. In insect diapause ferritin could limit exposure to oxygen radicals (of higher importance than in the hypoxic pupae) and/or contribute to the characteristic developmental arrest seen during diapause by restricting the iron availability for cell growth. Interestingly, our findings of higher ferritin abundance in diapausing larvae are in agreement with a previous report of diapause-dependent ferritin upregulation in copepods [43], which indicates a possibly widespread importance for diapause in arthropods. In this context, artemin, a ferritin homologue from *Artemia*, should be mentioned since it is also known to be up-regulated during diapause [44]. However, artemin does not bind iron and is thus unlikely to function in the same way as ferritin during diapause.

Heat shock proteins are proteins that rapidly change their expression patterns in response to an environmental shock and help prevent organismal damage. It is well established that *hsp* expression can change with diapause [3], [45].

In *Sarcophaga*, *hsp90* expression is high in non-diapausing and early diapause-destined individuals but drops significantly thereafter [46]. In our study HSP90 abundance was initially high in NDP6 and DP6 larvae and then dropped in DP7 and DP8. This overlap in expression patterns across species (*Nasonia* and *Sarcophaga*) and across metabolic levels (mRNA and protein) points to a conserved role during diapause (for a discussion of potential hsp functions during diapause see [46]). Similar to known expression trends for other *hsps*, we also detected increasing levels of protein essential for life (lefl, a HSP20 family member) during diapause. Consequently, it seems that mechanisms, which during normal development serve to protect the organism from damage, are co-opted for a novel life history trait, i.e. diapause. However, since diapause is often elicited by harsh conditions, it is unclear whether such proteins are necessary to trigger the diapause response, or if the change in their expression patterns is merely a consequence of environmental factors. Research on organisms with obligatory diapause should be able to shed light on this question.

Diapause can be maintained for several years [47] thus putting a metabolic strain on the organism. Therefore, one could expect that larvae increase their reserves in preparation for diapause. However, previous reports on insect storage proteins show that not all proteins classified as storage proteins show the same expression trends during diapause [48]–[53]. This heterogeneity of patterns is also reflected in our results.

In general, most of the storage proteins identified in our study (6 out of 7) seemed to accumulate in at least one of the diapause stages. Apolipophorins are able to bind and relocate lipids [54], [55]. Thus, their higher levels during diapause could be associated with increased lipid metabolism, which is a known diapause-related trait for some insects [6]. In this context, it is interesting to note that some peptides implicated in fat metabolism were conspicuously absent in the non-diapausing larvae of *Nasonia* [Figure 1 and supplementary table S1], which further corroborates a change of lipid metabolism with diapause in *Nasonia*.

On the other hand, GLX storage proteins and hexamerins could function as amino acid donors during diapause. In addition, some hexamerins are known or suspected to bind juvenile hormone, a major regulator of insect diapause [56], [57], and could thus play a role in controlling the duration of diapause. We found indications that the levels of a JH-degrading enzyme (JH epoxide hydroxylase) increase with time in diapause (data not shown because this protein barely missed our criteria for quantification). Juvenile hormone and its analogues have been reported to either induce or break diapause e.g. [58], [59]. In *Nasonia*, the effects of JH are unclear but it appears that, in agreement with our results, JH does not have a stimulatory effect on diapause in this species [20].

This study generated, with a single experiment, a multitude of candidate genes for early diapause, and identified potential physiological processes essential for diapausing in *Nasonia vitripennis*. This demonstrates how the availability of a whole genome sequence in combination with modern proteomic tools can significantly advance the study of the molecular changes underlying phenotypically plastic traits like diapause. Overall, we found major metabolic changes that confirm previous studies, but also reveal new insights into the physiology and regulation of developmental diapause in *Nasonia*, which showcases how non-gel shotgun proteomics can be used to map underpinnings of this syndrome.

The results of this study can now spur targeted studies on individual physiological processes or genes, which can eventually lead to a better understanding of the molecular mechanism underlying diapause in *Nasonia* in particular and invertebrates in general.

## Supporting Information

Table S1 Peptides identified in diapausing and non-diapausing *Nasonia* larvae combined under protein accession numbers. DP6-8: diapausing larvae collected at days 6-8. NDP6: non-diapausing larvae collected at day 6.
(0.33 MB XLS)

Table S2 Individual peptides identified in diapausing and non-diapausing *Nasonia* larvae.DP6-8: diapausing larvae collected at days 6–8. NDP6: non-diapausing larvae collected at day 6.
(13.01 MB XLS)
